# Prolonged efficacy of cefazolin in intraosseous regional prophylaxis for total knee arthroplasty: a rabbit model study

**DOI:** 10.1186/s12891-024-07238-y

**Published:** 2024-02-01

**Authors:** Jing-Yu Zhang, Ling-Chen Ye, Yu-bo Liu, Xiao Yu, Xiang-Xin Zhang, Guang-Xiang Chen, Ren-Jie Xu

**Affiliations:** Department of Orthopaedics, Suzhou Municipal Hospital, The Affiliated Suzhou Hospital of Nanjing Medical University, Suzhou, China

**Keywords:** Blood concentration, Cefazolin, Intraosseous regional administration, Periprosthetic joint infection, Total knee arthroplasty

## Abstract

**Background:**

A novel approach known as intraosseous regional administration (IORA) has emerged as a technique for delivering prophylactic antibiotics, and it results in higher tissue concentrations around the knee. It is hypothesized that IORA of cefazolin for antibiotic prophylaxis during total knee arthroplasty will result in sustained effective levels for a longer duration. The aim of the current study was to investigate temporal changes in peri-knee cefazolin blood concentrations after IORA of cefazolin.

**Methods:**

Twelve rabbits were randomly divided into two groups, with six rabbits in each group. In control group a single intravenous bolus injection of cefazolin (10 mL, 100 mg) was administered into the marginal ear vein. In experimental groupexperimental group the same dose of cefazolin was injected into the left tibial marrow cavity after tourniquet inflation at the base of the left thigh. Blood samples were collected periodically at different timepoints, and cefazolin concentrations were determined.

**Results:**

The intraosseous treatment resulted in significant differences in plasma cefazolin concentrations at all timepoints. Experimental group exhibited higher plasma cefazolin concentrations than control group.

**Conclusions:**

Cefazolin in intraosseous regional prophylaxis exhibits effectiveness in intraoperative antibiotic prophylaxis by maintaining concentrations above the minimum inhibitory concentration for extended durations, rather than relying solely on high concentrations.

## Background

Total joint arthroplasty is one of the most successful orthopedic procedures, but periprosthetic joint infection (PJI) remains a devastating complication that imposes a significant burden on patients and the healthcare industry [1, 2]. Perioperative prophylactic antibiotic administration has proven effective for reducing postoperative PJI [3].

A novel approach known as intraosseous regional administration (IORA) has emerged as a technique for delivering prophylactic antibiotics, and it results in higher tissue concentrations around the knee [4–6]. Both vancomycin and cefazolin have been used in previous studies. Notably however, cefazolin, as a time-dependent antibiotic, differs from concentration-dependent antibiotics such as vancomycin. Time-dependent antibiotics exhibit optimal bactericidal activity when drug concentrations are maintained above their minimum inhibitory concentration (MIC), typically at 2 to 4 times the MIC throughout the dosing interval. Unlike concentration-dependent antibiotics, higher concentrations of time-dependent antibiotics do not necessarily enhance the eradication of organisms.

Based on the above-described considerations it was hypothesized that IORA of cefazolin for antibiotic prophylaxis in total knee arthroplasty would result in sustained effective levels for a longer duration, but no currently available literature supports this hypothesis. The present study investigated temporal changes in peri-knee cefazolin blood concentrations after IORA.

## Methods

### Ethics approval

The study received approval from the Ethics Committee of Suzhou Municipal Hospital (approval number J-2022-063-H01). The study was conducted in accordance with the National Institutes of Health Guidelines and adhered to the Animal Research: Reporting In Vivo Experiments (ARRIVE) criteria.

### Rabbits

Twelve New Zealand albino rabbits weighing 4.0 ± 0.2 kg were used. All rabbits underwent a 1-week acclimatization period and were housed under standardized conditions, including a 12-hour light/dark cycle, a room temperature of 20 °C ± 2 °C, and a humidity level of 55% ± 5%. The rabbits had *ad libitum* access to food and water.

### Chemicals and reagents

Cefazolin (Huarun, Shenzhen, China) was procured from Suzhou Municipal Hospital. Titetamme and Zolazepam (Zoletil 50; Virbac, Carros, France), idazoxan hydrochloride 60 mg in 2 mL; Baite), and Xylazine hydrochloride (0.2 g in 2 mL; Baite, Chang Sha, China)were purchased from Chow Fung Veterinary Hospital.

### Experimental design

To induce sleep, a simultaneous intramuscular injection of 0.1 mL/kg Zoletil and 0.1 mL/kg xylazine was administered. When the rabbits were semi-conscious an additional 1/4 of the dose was given. At the end of the experiment wakefulness was induced by intramuscular injection of 0.1 mL/kg idazoxan.

The 12 rabbits were randomly divided into two groups of 6 rabbits, control group and experimental group. In control group a single intravenous bolus injection of cefazolin (100 mg in 10 mL) was administered into the marginal ear vein. In the experimental group, the equivalent dose of cefazolin was administered by injecting it into the marrow cavity of the left tibia using a bone marrow puncture needle (MN100 15 mm*15 G, Amathes, Suzhou, China) after inflating the tourniquet at the base of the left thigh. The tourniquet pressure was adjusted according to limb occlusion pressure monitored by ultrasound [7]. Sterile pre-heparinized 2-mL test tubes were used to collect blood samples from the subcutaneous vein of the left limb at periodic timepoints.

**Fig. 1:**
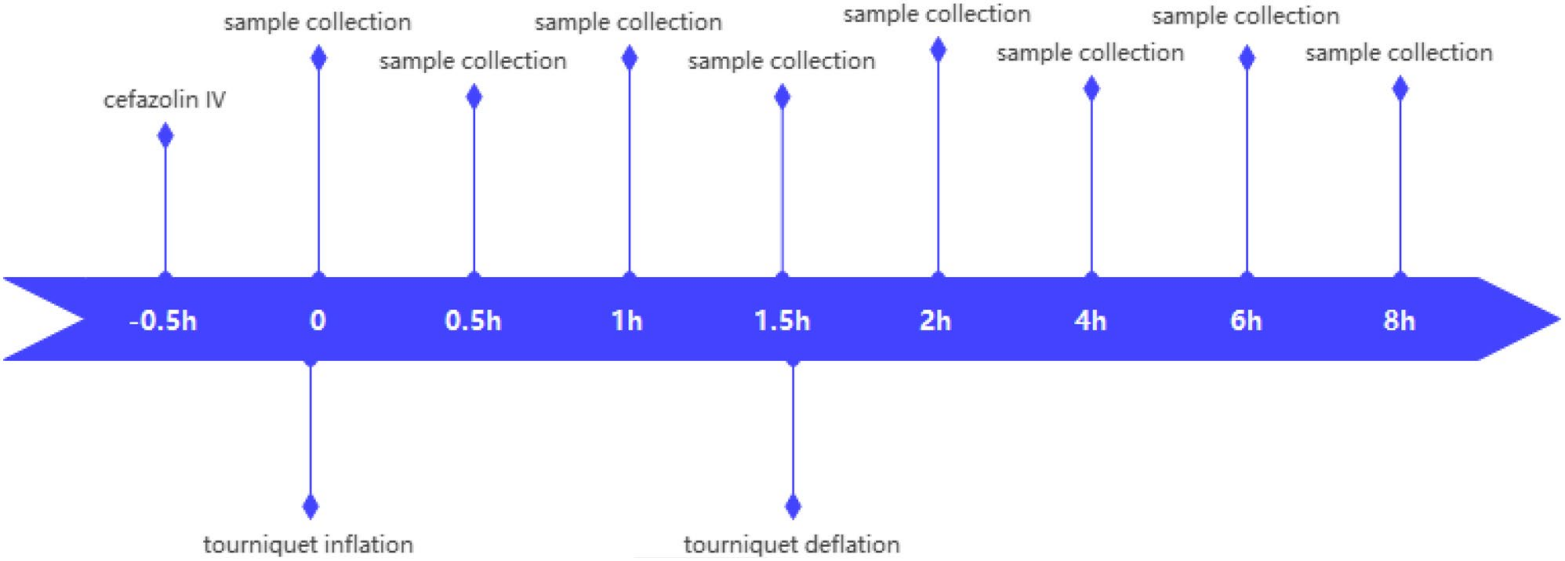
Blood collection timepoints in control group

In the control group, the tourniquet was inflated 0.5 h after administering antibiotics and remained in place for 1.5 h. Blood samples were collected at specific time points, including 0, 0.5, 1.0, 1.5, 2, 4, 6, and 8 h after tourniquet inflation. It is important to note that blood collection at 1.5 h after tourniquet inflation was conducted before deflating the tourniquet, as indicated in Fig. 1. Approximately 1 mL of blood was collected at each time-point. After centrifuging the blood samples at 4000 revolutions per min (rpm) for 10 min at 4 °C, the plasma was separated. Plasma samples were then transferred to 2-mL cryovials and stored at -20 °C, prior to determination of cefazolin concentrations via high-performance liquid chromatography (HPLC).

**Fig. 2:**
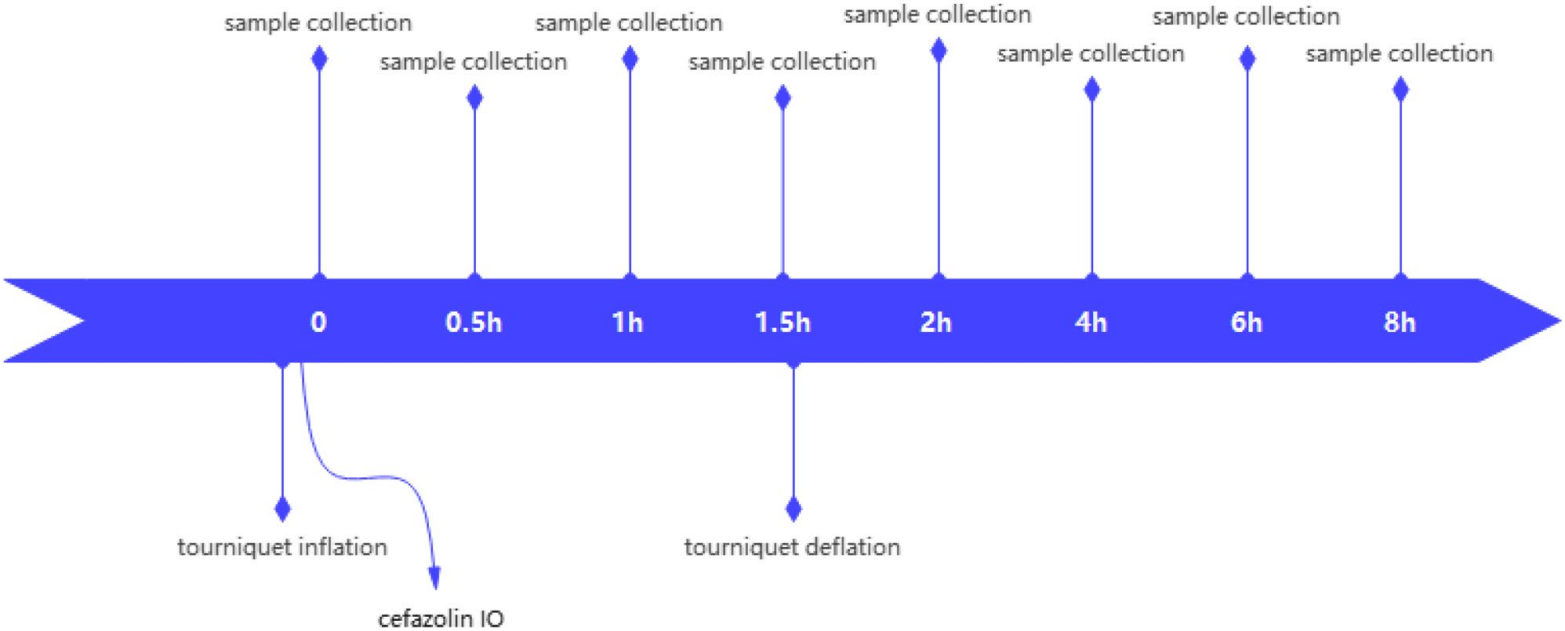
Blood collection timepoints in experimental group

In the experimental group, the same dose of cefazolin was injected into the left tibial marrow cavity after tourniquet inflation at the base of the left thigh. Blood samples were collected at specific time points, including 0, 0.5, 1.0, 1.5, 2, 4, 6, and 8 h after administering antibiotics. It is important to note that blood collection at 1.5 h after tourniquet inflation was conducted before deflating the tourniquet, as indicated in Fig. 2. The procedures used for sample collection, plasma separation, and storage were the same as those described with respect to control group.

### Extraction of plasma samples

To extract 300-µL plasma samples, deproteinization was conducted by adding 900 µL of acetonitrile for precipitation. The mixture was vortexed for 1 min then centrifuged at 15,000 rpm for 10 min at 4 °C. The clear supernatant was collected in 2mL Eppendorf tubes. A 50-µL aliquot of the resulting supernatant was injected into the HPLC system via an autosampler. Data integration was performed using EyouLabIVD software version 5.0.

### HPLC analysis of cefazolin concentration

**Fig. 3:**
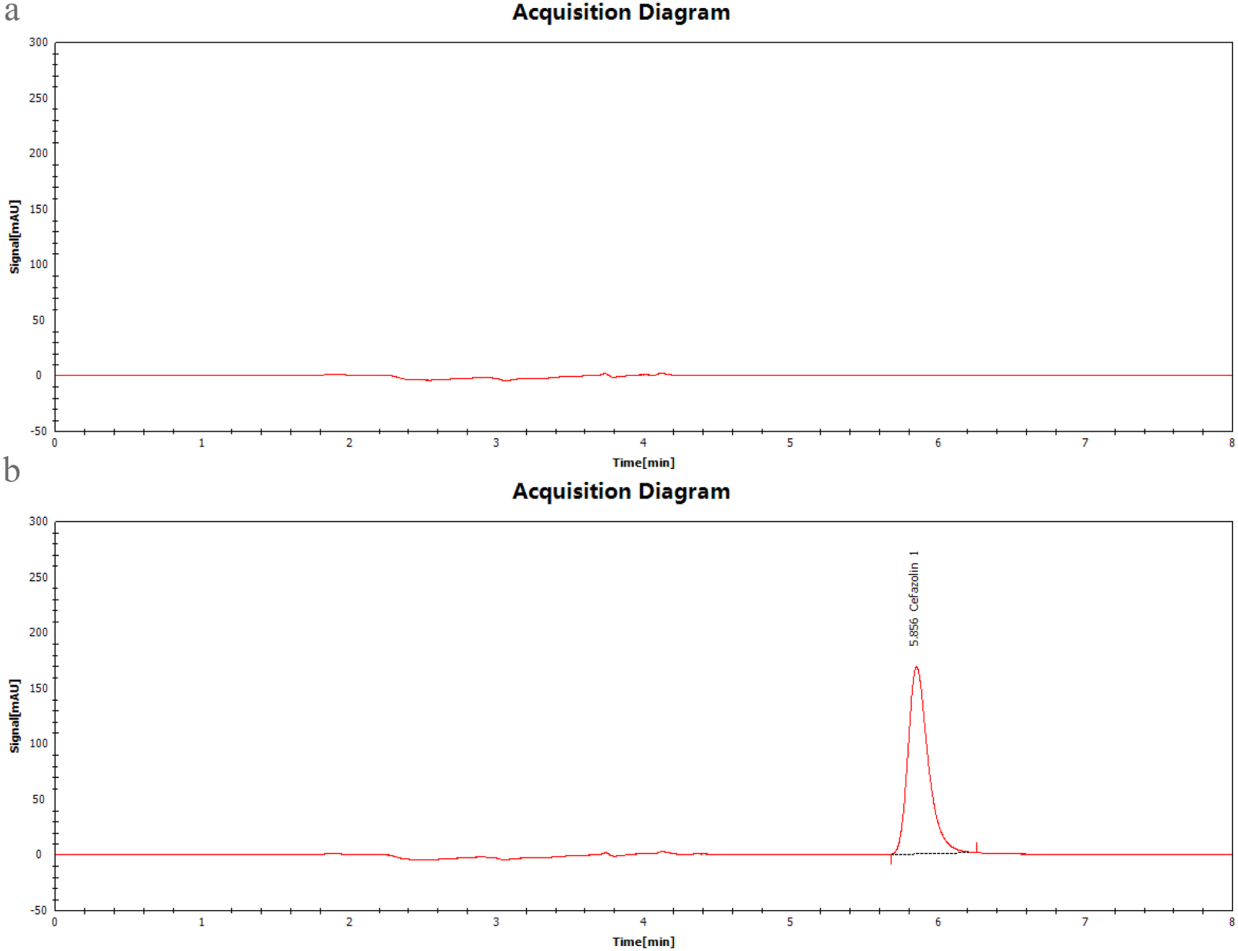
Chromatograms of (**a**) control plasma and (**b**) plasma spiked with cefazolin

For quantification of cefazolin in plasma samples an HPLC apparatus (Chromai, China) consisting of a UV detector (CMD-D10), gradient solvent delivery pump (CMD-P60), autosampler (CMD-A50CV), and thermostat (CMD-C10V6) was used. Chromatographic separation was achieved using a reverse-phase C18 analytical column (Lotus C18, 250.0 × 4.6 mm, 5 μm) at 40 °C. The mobile phase consisted of a mixture of 0.2% triethylamine (prepared in HPLC water, with formic acid adjusted to pH 4.0) and acetonitrile (80:20), which was filtered using a 0.45-µm filter (Millipore^®^, Merck Life Science Pvt. Ltd., Bangalore, India). The mobile phase was pumped into the column at a flow rate of 1.0 mL/min. Effluents were monitored at a wavelength of 280 nm using a UV detector (Fig. 3).

**Fig. 4:**
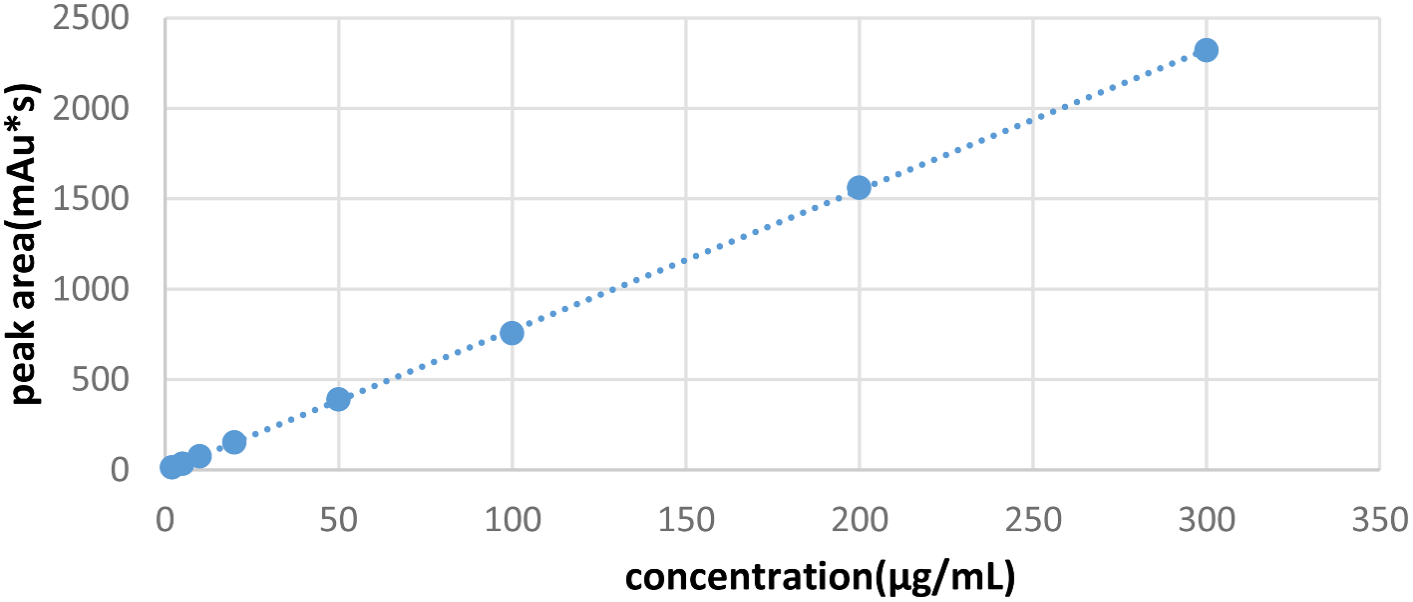
Standard calibration curve for cefazolin

For standardization, calibration samples of rabbit plasma containing cefazolin at concentrations of 2, 5, 10, 20, 50, 100, 200, and 300 µg/mL were prepared using rabbit plasma as the matrix. The samples were pretreated and quantified using the external standard method with a Chromai Voyager 3500 HPLC system. The standard calibration curve for cefazolin exhibited linearity within the concentration range of 2 to 300 µg/mL. Cefazolin quantification in plasma samples was performed by referencing the resulting standard curve. The assay demonstrated sensitivity, reproducibility, and linearity, with a mean correlation coefficient (R2) > 0.9999 (Fig. 4). Plasma concentration-time curves were plotted using Microsoft Excel (Version 2013, Redmond, Washington, DC, USA).

### Statistical analysis

Statistical analysis was performed using SPSS 19.0 software (IBM Corp, Armonk, NY, USA). Continuous variables are presented as mean ± SD. Student’s *t*-test was used to compare means between the two groups. *p* < 0.05 was considered statistically significant.

## Results

**Fig. 5:**
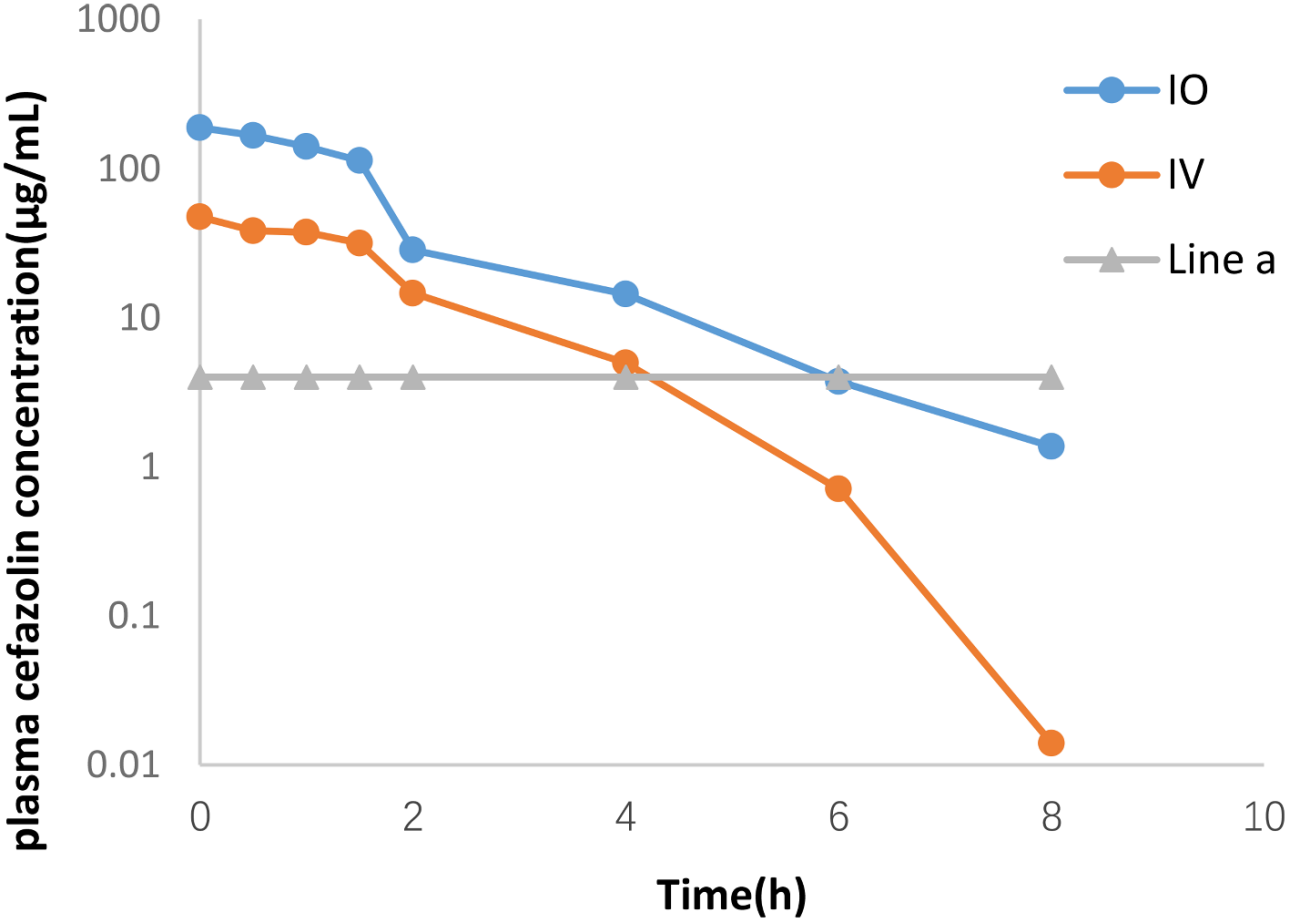
Semi-logarithmic plot of plasma cefazolin concentrations by time after intravenous administration or intraosseous administration. Line a = 4 µg/mL (four times the MIC90 of MSSA)

Intraosseous treatment resulted in significant differences in cefazolin plasma concentrations at all timepoints. Experimental group exhibited higher plasma cefazolin concentrations than control group (Table 1). Semi-logarithmic plots of plasma cefazolin concentrations after intravenous (IV) administration and intraosseous administration are shown in Fig. 5. According to Yamada’s [8] definition of adequate concentration for methicillin-sensitive *Staphylococcus aureus* (MSSA) (4 µg/mL, four times the MIC90 of MSSA), experimental group maintained a concentration above the adequate level for approximately 6 h, whereas control group maintained it for 4 h.

**Table 1:** Cefazolin plasma concentrations

Time (h)	Control group (µg/mL)	Experimental group (µg/mL)
0	47.529 ± 7.333	188.51 ± 26.293*
0.5	38.357 ± 8.782	167.273 ± 16.758*
1	37.423 ± 7.651	140.724 ± 19.654*
1.5	31.712 ± 7.118	113.416 ± 9.666*
2	14.642 ± 3.169	28.482 ± 4.341*
4	4.973 ± 2.784	14.409 ± 4.072*
6	0.711 ± 0.823	3.743 ± 0.586*
8	0.014 ± 0.034	1.371 ± 0.175*

*p < 0.05

## Discussion

The results of the current study indicate that when used for intraoperative antibiotic prophylaxis in total knee arthroplasty, higher blood cefazolin concentrations are sustained for an extended period. This is particularly advantageous with respect to timedependent antibiotics such as cefazolin, because it enhances the bactericidal activity of the drug.

According to the Clinical Practice Guidelines for Antimicrobial Prophylaxis in Surgery [9], cefazolin is recommended as the first choice for antimicrobial prophylaxis in patients undergoing total hip and knee arthroplasty. Yamada et al. [8] reported that conventional systemic prophylactic cefazolin dosing achieved the minimum inhibitory concentration for MSSA, but fell short of providing sufficient tissue concentrations against coagulase-negative staphylococci. Conversely, in overweight patients standard antibiotic doses can lead to underdosing, increasing the risk of PJI [10]. In 2013 Young et al. [4] proposed a novel method to enhance local antibiotic concentrations in knee arthroplasty. IORA involves injecting a prophylactic antibiotic into the proximal tibial metaphyseal bone, creating a Biers block of antibiotic to the limb with an inflated tourniquet on the leg. Within seconds the antibiotic from the tibial metaphyseal bone flows directly into the limb’s venous system, filling the limb below the tourniquet. The efficacy of the technique with respect to achieving higher tissue concentrations is well established [4–6, 11, 12], and it has been shown to effectively reduce the incidence of PJI in cases of total knee arthroplasty [13, 14]. Cefazolin is a time-dependent intraoperative prophylactic antibiotic that is amenable to maintenance of concentrations above its MIC for extended durations, rather than relying solely on high initial concentrations.

The current study had some limitations. The knee replacement procedure simulated did not include a bleeding washout, which is typically performed during human operations, and can potentially lower cefazolin blood concentrations. It is important to note that human antibiotic doses may differ from those used in animal experiments, leading to variations in blood concentrations. Third, the use of cement in knee replacements, which cures through an exothermic reaction, is indeed relevant to the IORA technique. The exothermic reaction that occurs during cement curing can indirectly affect the effectiveness of IORA. The heat generated during cement curing has the potential to degrade antibiotics or alter their pharmacokinetics, thereby impacting their concentration and distribution within the bone. Therefore, it is essential to carefully consider the timing and technique of antibiotic administration in relation to the cement curing process to ensure optimal antibiotic delivery and efficacy.

## Conclusions

Cefazolin in intraosseous regional prophylaxis exhibits effectiveness in intraoperative antibiotic prophylaxis by maintaining concentrations above the minimum inhibitory concentration for extended durations, rather than relying solely on high concentrations.

## Data Availability

The datasets used and analyzed during the current study are available from the corresponding author on reasonable request.

## Abbreviations

HPLC High: performance liquid chromatography
MIC: Minimum inhibitory concentration
MSSA: Methicillin-sensitive Staphylococcus aureus
PJI: Periprosthetic joint infection

## References

[1] Urquhart DM, Hanna FS, Brennan SL, Wluka AE, Leder K, Cameron PA (2010). Incidence and risk factors for deep surgical site infection after primary total hip arthroplasty: a systematic review. J Arthroplasty.

[2] Lum ZC, Natsuhara KM, Shelton TJ, Giordani M, Pereira GC, Meehan JP (2018). Mortality during total knee periprosthetic joint infection. J Arthroplasty.

[3] AlBuhairan B, Hind D, Hutchinson A (2008). Antibiotic prophylaxis for wound infections in total joint arthroplasty: a systematic review. J Bone Joint Surg Br.

[4] Young SW, Zhang M, Freeman JT, Vince KG, Coleman B (2013). Higher cefazolin concentrations with intraosseous regional prophylaxis in TKA. Clin Orthop Relat Res.

[5] Young SW, Zhang M, Freeman JT, Mutu-Grigg J, Pavlou P, Moore GA (2014). The Mark Coventry Award: higher tissue concentrations of Vancomycin with low-dose intraosseous regional versus systemic prophylaxis in TKA: a randomized trial. Clin Orthop Relat Res.

[6] Young SW, Zhang M, Moore GA, Pitto RP, Clarke HD, Spangehl MJ (2018). The John N. Insall Award: higher tissue concentrations of Vancomycin achieved with intraosseous regional prophylaxis in revision TKA: a Randomized Controlled Trial. Clin Orthop Relat Res.

[7] Papalia R, Zampogna B, Franceschi F, Torre G, Maffulli N, Denaro V (2014). Tourniquet in knee surgery. Br Med Bull.

[8] Yamada K, Matsumoto K, Tokimura F, Okazaki H, Tanaka S (2011). Are bone and serum cefazolin concentrations adequate for antimicrobial prophylaxis?. Clin Orthop Relat Res.

[9] Bratzler DW, Dellinger EP, Olsen KM, Perl TM, Auwaerter PG, Bolon MK (2013). Clinical practice guidelines for antimicrobial prophylaxis in surgery. Surg Infect.

[10] Rondon AJ, Kheir MM, Tan TL, Shohat N, Greenky MR, Parvizi J (2018). Cefazolin Cefazolin prophylaxis for total joint arthroplasty: obese patients are frequently underdosed and at increased risk of periprosthetic joint infection. J Arthroplasty.

[11] Chin SJ, Moore GA, Zhang M, Clarke HD, Spangehl MJ, Young SW (2018). The AAHKS clinical research award: intraosseous regional prophylaxis provides higher tissue concentrations in high BMI patients in total knee arthroplasty: a randomized trial. J Arthroplasty.

[12] Spangehl MJ, Clarke HD, Moore GA, Zhang M, Probst NE, Young SW (2022). Higher tissue concentrations of Vancomycin achieved with low-dose intraosseous injection versus intravenous despite limited tourniquet duration in primary total knee arthroplasty: a randomized trial. J Arthroplasty.

[13] Park KJ, Chapleau J, Sullivan TC, Clyburn TA, Incavo SJ, Chitranjan S. Ranawat Award: intraosseous vancomycin reduces periprosthetic joint infection in primary total knee arthroplasty at 90-day follow-up. Bone Jt J. 2021;103-B:13 – 7. 10.1302/0301-620X.103B6.BJJ-2020-2401.R1. 10.1302/0301-620X.103B6.BJJ-2020-2401.R1 34053300

[14] Parkinson B, McEwen P, Wilkinson M, Hazratwala K, Hellman J, Kan H (2021). Intraosseous regional prophylactic antibiotics decrease the risk of prosthetic joint infection in primary TKA: a multicenter study. Clin Orthop Relat Res.

